# The Phenomena of Naturopathic Practitioner: Predictors of a High Patient Throughput

**DOI:** 10.1155/2017/9758326

**Published:** 2017-11-02

**Authors:** Katja Goetz, Stefanie Kattge, Jost Steinhäuser

**Affiliations:** Institute of Family Medicine, University Hospital Schleswig-Holstein, Campus Luebeck, Ratzeburger Allee 160, 23538 Luebeck, Germany

## Abstract

*Objective. *The aim of the current study was to evaluate which factors predicted a high patient throughput to add more evidence to the phenomena of naturopathic practitioners.* Methods. *The cross-sectional study was based on a questionnaire with a sample of 1,096 naturopathic practitioners in the German Federal State of Schleswig-Holstein. Besides, sociodemographic data and practice characteristics topics like job satisfaction and feeling for the job were evaluated. This was supplemented with an evaluation of patient traits which were perceived as challenging. Descriptive statistics and binary regression analysis were computed to identify potential predictors to a high patient throughput.* Results. *A response rate of 29.4% (322/1096 participants) was observed for the study. In general, our sample of the naturopathic practitioners was very satisfied with their job (mean = 6.38). Naturopathic practitioners described that 40% of their patients are challenging. The highest rate was for “aggressive patients.” A high patient throughput was predicted with a higher satisfaction rate with the “opportunity to use abilities” and more direct contact with the patient.* Conclusions. *Therapeutic freedom and time with patients are important factors which are accountable for a high patient throughput. Moreover, our study provides evidence for the understanding of the phenomena of naturopathic practitioners.

## 1. Introduction

As in other western countries, Complementary and Alternative Medicine (CAM) has become more and more popular for the population in Germany during the last years [1]. In the year 2012 over 63% of the inhabitants had experience with a treatment method which is attributed to CAM [2]. This phenomenon was also observed in countries such as the United States, Australia, and Switzerland [3–5]. Internationally, CAM is provided by physicians but also by nonmedical practitioners who are named as naturopathic practitioners (Heilpraktiker). However, a clear definition about the area of responsibility offered by naturopathic practitioners does not exist yet. Consequently, there is a huge variability concerning national regulatory management which implicated that a comparison across the European Union is almost impossible [6].

The situation in Germany for becoming a naturopathic practitioner is as follows. These nonmedical practitioners are organized in different professional associations. Currently they have no regulated health workforce group which implies that each association offers its own curriculum for qualification. The qualification as a naturopathic practitioner is linked to individual study or through a privately financed naturopathic practitioner school (Heilpraktikerschule). The primary requirements for becoming a naturopathic practitioner are regulated within the naturopathic practitioner law (Heilpraktikergesetz) [7]. A person who aims for becoming a naturopathic practitioner has to be at least 25 years old and pass an examination, which is conducted by the local Public Health Office within each federal state in Germany. After passing the examination, they are allowed to practice independently (self-employed) in their own practice. The number of naturopathic practitioners is continuously rising in Germany. The statistics report from the German Federal Statistical Office for the year 2000 shows over 13,000 naturopathic practitioners in Germany, while this figure reaches 35,000 for the year 2011. On the other hand, the number of medical practitioners as of 2011 was 342,000 [8]. This results in a ratio of about 1 : 10.

The naturopathic practitioners offered a broad range of treatment methods from para-medicinal treatment such as bioresonance therapy or iris diagnosis to classic natural remedy methods such as phytotherapy or balanced nutrition [9]. The naturopathic practitioner has a direct treatment contract with the patient and has therapeutic freedom in his/her practice. These are important deferrals concerning the use of conventional medicine by patients and could contribute to the naturopathic practitioners feeling positive about their job. A qualitative study, which mainly depends on the health-professional and patient relationship, found that naturopathic practitioners feel more positive about job-related satisfaction than conventional practitioners [10].

Moreover, there exists some knowledge regarding the kind of healthcare issues that predict a higher use of CAM [11]. However, to our knowledge, there is no literature regarding factors that predict a high patient throughput in naturopathic practitioners. Therefore, the aim of this study was to evaluate which factors predicted a high patient throughput to add more evidence to the phenomena of naturopathic practitioners.

## 2. Methods

The observational study was based on a questionnaire survey with naturopathic practitioners in the German Federal State of Schleswig-Holstein.

### 2.1. Participants

As there is no central register of all naturopathic practitioners, participants were recruited via two online-telephone-books [12, 13]. Naturopathic practitioners for psychotherapy, who were also listed in the phone-books, were excluded as well as those who treat animals or both humans and animals. Doing so, 1,096 naturopathic practitioners in the German Federal State of Schleswig-Holstein were identified and invited to participate in the questionnaire survey. Data was collected from September 2015 until January 2016. As an alternative to completing the questionnaire, there was a short-response sheet for nonparticipants, which could be completed and returned instead. The nonresponse-sheet evaluated only sociodemographic data like age and gender and reasons for nonparticipation. The return of the anonymous paper-based questionnaire was classified as informed consent. No reminders were sent out. Because this was an explorative study, no power calculation was determined.

### 2.2. Measures

Sociodemographic and practice characteristics were measured in the questionnaire including gender, age, duration of employment, and the location of the practice (rural or urban). Moreover, different practice characteristics were evaluated including patient contact per week, the time of direct patient contact per week, the hours of communication with patients per week, the time of practice activities per week, and the time of administrative tasks per week. Job satisfaction among naturopathic practitioners was measured by the German version of the modified Warr-Cook-Wall job satisfaction scale (WCW-scale), developed by Warr et al. [14, 15]. On a 7-point Likert scale naturopathic practitioners could rate their response between “1 = extreme dissatisfaction” and “7 = extreme satisfaction.” The feeling for the job was measured with two items. These were “the job contributes to a meaningful life” and “I look forward to working when I wake up in the morning.” These items were rated on a 5-point Likert scale between “1 = fully disagree” and “5 = fully agree” and were developed by Professor J. Fischer, Institute of Public Health, Mannheim, Germany, but not published. Furthermore, naturopathic practitioners were asked to estimate the percentage of challenging traits of patients in their practice. Therefore, a questionnaire was developed consisting of 8 traits such as aggressive, unfriendly, and anxious. The traits chosen were defined by a selective literature search [16, 17]. Naturopathic practitioners were asked to assess the challenges they perceive regarding these traits on a 10-point Likert scale. They could choose between “1 = not challenging at all” and “10 = very challenging.” A high mean score indicates high challenge for the specific traits. The questionnaire can be requested from the authors of this manuscript.

Furthermore, an extra sheet, nonresponder survey, was supplemented to the questionnaire for those who did not want to participate in the survey. The nonresponder survey includes gender, age, and reasons for nonresponding.

### 2.3. Data Analysis

The descriptive analysis was performed with SPSS version 24.0 (SPSS Inc., IBM). Firstly, a descriptive analysis was undertaken to determine characteristics of the study population. Furthermore, descriptive analysis of the job satisfaction scale, feeling for the job, and challenging traits of clients was conducted. The means and standard deviations of these three aspects were reported. Furthermore, a binary logistic regression analysis was performed with the binary variable “patient contact per week.” The variable was split under consideration of the median. Therefore, the category “1” was for more than 15 patients per week and “0” for 15 patients and fewer. The binary variable “patient contact per week” was the outcome variable and descriptive characteristics, job satisfaction, feeling for the job, and challenging traits of clients were handled as explanatory variables. The Hosmer-Lemeshow test was used to evaluate the suitability of the logistic regression model and the Nagelkerkes R-Quadrat was used for the explained variance of the model [18, 19]. Missing data <10% was negligible for the data analysis. An alpha level of *p* < 0.05 was used for tests of statistical significance.

### 2.4. Ethical Approval

The study was approved by the ethics committees of the University of Luebeck (number 15-265). Completion of the survey was voluntary and anonymous. No additional data were evaluated.

## 3. Results

Out of 1,096 questionnaires handed out, 322 questionnaires from naturopathic practitioners were returned (response rate 29.4%). 60 (18.6%) of the returned questionnaires were the short-response sheets, here called the nonresponders.  Table 1 presents the sociodemographic and practice characteristics of participants (*n* = 262) and nonresponders (*n* = 60). More than 80% of the responding naturopathic practitioners were female. The mean age was 53 years (SD = 9.5). The mean patient contact per week was 20.4. In our sample, 55% of the naturopathic practitioners stated that they treated 15 patients or fewer per week, while 42% of our participants treated more than 15 patients per week. The sample consisted of 51.1% participants working in rural areas. More than 75% of the nonresponding naturopathic practitioners were female and had a mean age of 57.8 years (SD = 9.7). The most common reason for nonresponding was that they were principally not willing to participate in surveys.

### 3.1. Evaluation of Job Satisfaction and Feeling for the Job

The evaluation of job satisfaction and feeling for the job is presented in Table 2. A high satisfaction rate was observed for the items “satisfaction with freedom of working method” (mean = 6.79) and “satisfaction with amount of variety in job” (mean = 6.38). A less satisfaction rate was found for the item “satisfaction with income/professional fees” (mean = 4.56). The overall satisfaction with the job was quite good with a mean of 6.38. A high agreement was found for the feeling for the job in both items.

### 3.2. Challenging Traits

Naturopathic practitioners perceived 40% of their patients as challenging. Different challenging patient traits are presented in Table 3. Patients who were observed as aggressive and less compliant were ranked as highly challenging with a mean of 6.53 and 6.43, respectively. In contrast patients who were observed as anxious and those with a lot of questions were ranked as less challenging with a mean of 3.68 and 3.91, respectively.

### 3.3. Predictors of a High Patient Throughput

The binary regression model regarding the treatment of more patients per week showed four factors with a Nagelkerke *R*^2^ of 0.797 (Hosmer-Lemeshow test *p* = 0.43) and is presented in Table 4. Only significant results were shown. More patients per week was significantly associated with an increasing satisfaction with “the opportunity to use abilities” (odds ratio (OR) = 2.20, confidence interval (CI) = 1.18, 4.12), less minutes of contact per patient (OR = 0.90, CI = 0.86, 0.94), more direct contact with patients (OR = 1.27, CI = 1.14, 1.41), and more hours of communication with the patients per week (OR = 1.38, CI = 1.05, 1.80).

## 4. Discussion

The aim of this study was to evaluate the factors that predicted a high patient throughput by naturopathic practitioners. Our results showed that a high proportion of participants, naturopathic practitioners, have worked for a long time as naturopathic practitioners. Moreover, the participating naturopathic practitioners spent significantly more time with their patients than the physicians did [20]. We evaluated a mean of 60 minutes of patient contact time compared with a mean of 9 minutes which was found for German physicians within the Commonwealth fund survey [20]. Considering that more and more patients visit a naturopathic practitioner, it can be assumed that they are dissatisfied with the consultation time offered by conventional medical practitioners. Studies showed that the consultation time is an important aspect for patients' satisfaction with their care and could influence compliance and adherence [21, 22]. The fact that naturopathic practitioners have a very positive feeling for the job and are very satisfied in doing the job, with the exception of income, has already been discussed elsewhere [23]. However, the relationship of job satisfaction, challenging patient traits, and a high patient throughput by the professional group of naturopathic practitioners are unknown.

Naturopathic practitioners perceived more of their patients as challenging than in general practice care. For general practice care, it was found that only 16% of the patients are perceived as challenging [24, 25]. The evaluation of different patient traits from the perspective of naturopathic practitioners in our sample showed that “aggressive patients” have the highest rate from the list of challenging traits which is comparable to an interview study with family physicians. This study identified “aggressive patients” as the most difficult ones [26].

The binary regression analysis showed that a higher number of patients is strongly predicted with higher satisfaction concerning “opportunities to use abilities.” In contrast to conventional medicine, naturopathic practitioners could offer a broad range of different therapy methods from CAM to their patients in combination with freedom of the usage of different methods [27] whereby clear communication and explanation about the treatment procedures play a central role. Moreover, communication and time within direct patient contact are observed as strong predictors for a high patient throughput. A study which compared the visit length in complementary medicine and general practice evaluated a longer time for visit in complementary medicine [28]. Different studies about naturopathic practitioners show that this professional group state that they consider patients as a whole person [29, 30]. It could be assumed that this would be one explanation why naturopathic practitioners are a frequently contacted professional group. Maybe specialist physicians such as family physicians should emphasize more on the holistic way of practicing. Another dissociation from conventional medicine is its use of evidence-based medicine (EBM) which is not a priority in naturopathy, because it could lead to restriction in offered treatment methods [30]. This qualitative study also showed that naturopathic practitioners perceived EBM as harmful to their image [30].

Our study provides an important contribution to the phenomena of naturopathic practitioners, especially to the frequently contacted naturopathic practitioners. All participating practitioners come from one federal state in Germany. Therefore, the findings could be tentative and it is not possible to determine cause-and-effect relationships. A strength of the study was the nonresponder evaluation. Only naturopathic practitioners were involved in this study; the perspective of patients' reasons for encounter and the perception of work offered by naturopathic practitioners should be a crucial part of further studies. The participation in this study was voluntary. Therefore, a potential selection bias is indicated. Finally, this was a cross-sectional study, and thus, we must be cautious to derive causal links from these findings.

## 5. Conclusions

The “opportunity to use abilities” and enough communication time with the patient are two important factors which are accountable for a high patient throughput in naturopathy. Our results could help in understanding the phenomena of naturopathic practitioners which is important for healthcare in general but also for healthcare professionals from conventional medicine. However, there is a research gap concerning the professional group of naturopathic practitioners. It would be advisable to intensify research within health services research to understand the work of naturopathic practitioners and its effects on patients' health.

## Figures and Tables

**Table 1:** Sociodemographic characteristics of participants and nonparticipants.

Characteristics^#^	Responder (*n* = 262)	Nonresponder (*n* = 60)
Gender; *n* (%)		
male	48 (18.3%)	10 (16.7%)
female	211 (80.5%)	45 (75.0%)
Age, years; mean (SD)	53 (9.5)	57.8 (9.7)
Period of employment; *n* (%)		
<5 years	42 (16.0%)	2 (3.3%)
5 and 10 years	65 (24.8%)	18 (29.9%)
11 and 20 years	81 (30.9%)	15 (25.1%)
>20 years	71 (27.1%)	16 (26.8%)
Patient contact per week; mean (SD)	20.4 (20.3)	
Average time of contact per patient, minutes; mean (SD)	60.3 (22.6)	
Overall time of direct contact with patients per week, hours; mean (SD)	17.2 (11.7)	
Average hours of communication per week with the patients; mean (SD)	3.4 (3.4)	
Average time of practice activities per week; mean (SD)	25.4 (15.2)	
Average time of administrative tasks per week; mean (SD)	4.8 (4.3)	
Location of practice, *n* (%)		
Rural	123 (51.1%)	
Urban	134 (46.9%)	

^#^ *n* varies due to missing values; SD: standard deviation.

**Table 2:** Descriptive statistics of job satisfaction and feeling for the job of naturopathic practitioners (*n* = 262).

	Mean (SD)	CI 95%
*Aspects of job satisfaction* ^*∗*^		
Physical working condition	5.84 (1.29)	5.62–6.01
Freedom of working method	6.79 (0.73)	6.60–6.86
Colleagues and fellow workers	5.61 (1.31)	5.41–5.80
Recognition for work	5.86 (1.27)	5.63–6.03
Amount of responsibility	5.96 (1.21)	5.79–6.15
Income/professional fees	4.56 (1.61)	4.34–4.81
Opportunity to use abilities	6.23 (1.18)	6.04–6.38
Hours of work	5.48 (1.51)	5.21–5.66
Amount of variety in job	6.38 (0.95)	6.18–6.49
Overall job satisfaction	6.38 (0.97)	6.20–6.50

*Feeling for the job* ^#^		
The job contributes to a meaningful life	4.75 (0.59)	4.67–4.82
I look forward to working when I wake up in the morning	4.49 (0.67)	4.41–4.57

^*∗*^Range from 1 “extreme dissatisfaction” to 7 “extreme satisfaction”; ^#^range from 1 “fully disagree” to 5 “fully agree”; SD: standard deviation; CI: confidence interval.

**Table 3:** Descriptive statistics of challenging traits of patients from the perspective of naturopathic practitioners.

Characteristic of patients^*∗*^	Mean (SD)	CI 95%
Aggressive	6.53 (3.28)	6.12–6.94
Limited compliance	6.43 (3.05)	6.05–6.80
Demanding	5.76 (2.64)	5.43–6.08
Unfriendly	5.32 (3.15)	4.93–5.71
Anancastic	4.95 (2.74)	4.62–5.29
Critical	4.39 (2.44)	4.09–4.69
Person with a lot of questions	3.91 (2.51)	3.61–4.22
Anxious	3.68 (2.55)	3.36–3.99

^*∗*^Range from 1 “not challenging at all” to 10 “very challenging”; SD: standard deviation; CI: confidence interval.

**Table 4:** Predictors of a high patient throughput: a binary-logistical regression model.

Variables	OR (95% CI)	*p* value
Satisfaction with the opportunity to use abilities	2.20 (1.18–4.12)	0.01
Average time of contact per patient, minutes	0.90 (0.86–0.94)	<0.01
Overall time of direct contact with patients per week, hours	1.27 (1.14–1.41)	<0.01
Average hours of communication per week with the patients	1.38 (1.05–1.80)	0.02

OR: odds ratio; 95% CI: confidence interval.
